# Host-guest mediated sensing of biologically relevant small molecules using supramolecular nanoassembly

**DOI:** 10.1186/1755-8166-7-S1-P80

**Published:** 2014-01-21

**Authors:** Alok Pandya, Pinkesh G Sutaria, Anand Lodha, Heena Goswami, Shobhana K Menon

**Affiliations:** 1 Institute of life Sciences, School of Science and Technology, Ahmedabad University, Ahmedabad, -380 009, Gujarat, India; 2 Department of Chemistry, School of Sciences, Gujarat University, Ahmedabad, 380 009, Gujarat, India

## Background

A high concern for human health and safety has motivated dynamic research on the potential impact of transition metal ions or molecule and their toxic effects. Thus, selective detection of biologically relevant molecule have enormously gained its attention due to involvement in a variety of fundamental environmental and biological process in organism because its deficiency and excess can induce a variety of diseases. Therefore, biomolecule detection have received a great deal of study. Here, we have designed an efficient strategy using supramolecular nanoassembly to detect biologically relevant small molecules with high specificity and selectivity and applicable to the biological milieus.

## Method

In our method, we designed a microwave assisted new promising approach using Silver (Ag) and Gold (Au) nanoparticles (NPs) based colorimetric sensing system (ANCSS) which form calix[4]arene- functionalized Ag and Au nanoprobe complex (CX-AgNPs/AuNPs) for the detection of biologically relevant small molecules such as ferric ion and glucose in water.

## Result

Driven by the need to detect trace amounts of Fe^3+^ and Glucose from blood samples, a molecular receptor based on calix[4]arene functionalized AuNPs/AgNPs was designed which proficiently and selectively recognizes glucose and Fe^3+^ in nanomolar level from aqueous solution with excellent discrimination against other heavy metals and biomolecule. The assembly was characterized by, DLS, UV-Vis, FT-IR, ESI-MS and ^1^H NMR spectrometry which demonstrates the higher binding affinity for Fe^3+^ and Glucose via weak forces. It is easy to operate and, most importantly, it exhibits fast response time (<80 s) and has long shelf-life (>4 weeks).

## Conclusion

The calix[4]arene functionalized Ag or AuNPs are able to selectively detect Fe^+3^ and glucose from aqueous medium and even from human blood. The key to the successful formation of this strategy is multi binding site of functionalized calix[4]arene which sensitively and selectively target to small molecules. The developed functionalized calixarene-Ag or AuNPs based human heme and gluocse biosensor has been proved to be a simple, reliable and accurate which can also indirectly assist to medeco-legal system for the routine investigation process.

